# Breaking Therapeutic Inertia With Alirocumab in an 80-Year-Old Patient With Severe Hypercholesterolemia: A Case Report

**DOI:** 10.3389/fmed.2021.699477

**Published:** 2021-07-07

**Authors:** Angela Dardano, Giuseppe Daniele, Giuseppe Penno, Roberto Miccoli, Stefano Del Prato

**Affiliations:** Section of Diabetes, Department of Clinical and Experimental Medicine, University of Pisa, Pisa, Italy

**Keywords:** LDL cholesterol, PCSK9 inhibitors, alirocumab, elderly, case report, therapeutic inertia, hypercholesterolemia

## Abstract

**Background:** Therapeutic inertia, defined as the failure to initiate or intensify therapy in a timely manner as per evidence-based clinical guidelines, is an important barrier limiting optimal care in the elderly. Therefore, overcoming therapeutic inertia is the core challenge when dealing with geriatric patients.

**Case Description:** The patient was an 80-year-old man that attended our Outpatient Lipid Clinic (Pisa University Hospital) because of persistent high LDL cholesterol (LDLc) levels in a setting of a statin contraindication. He underwent five percutaneous coronary angioplasties with drug-eluting stents. In 2014, upon starting treatment with rosuvastatin for LDLc level of 7.59 mmol/L, the patient was admitted to the Emergency Room for a presumptive diagnosis of rhabdomyolysis (creatine kinase 6685 U/L) secondary to statin. Patient developed acute kidney injury treated with dialysis. After resolution, he was discharged with ezetimibe (10 mg daily). This treatment however failed to effectively reduce LDLc levels that ranged between 5.9 and 6.6 mmol/L for the ensuing 4-years. In 2018, at the time of our evaluation, in consideration of the age, we performed a comprehensive geriatric assessment that showed good functional and mental status supporting a reliable treatment with a proprotein convertase subtilisin–kexin type 9 inhibitor. Therefore, alirocumab was prescribed as add-on to ezetimibe. At 24-month follow-up, the geriatric assessment showed no significant changes, and alirocumab was well-tolerated. LDLc was 82% lower as compared to baseline values (from 6.6 to 1.2 mmol/L).

**Conclusions:** This report describes a case of therapeutic inertia despite a very high-risk profile. It is also instrumental in highlightening that appropriate intensification of therapy in an elderly patient at high cardiovascular risk, by means of a patient-centered approach, may allow reaching therapeutic targets and overcoming the condition of therapeutic inertia.

## Introduction

The original concept of clinical inertia was highlighted by Phillips et al. (1) in 2001 who defined it as the failure to initiate or intensify therapy in a timely manner as per evidence-based clinical guidelines, thus emerging as an important barrier limiting optimal care in patients with chronic diseases such as type 2 diabetes mellitus (2), hypertension (3), dyslipidemia (4), coronary artery disease (5), and heart failure (6). Most of the literature on clinical inertia relate to failure to introduce appropriate therapies when treatment goals are not being met. However, in view of the wider application of the term, recent positions suggest that clinical inertia should be reserved for “lack of adherence to guideline recommendations when appropriate to do so,” with therapeutic inertia being used for “failure to advance therapy or to de-intensify therapy when appropriate to do so” (7). For our purpose we have agreed to use the term therapeutic inertia. Here we report a case of therapeutic inertia concerning an 80-year-old patient with high cholesterol levels and coronary ischemic heart disease. The patient was not treated with statins because of a previous episode of rosuvastatin-related rhabdomyolysis and other lipid-lowering drugs (i.e., proprotein convertase subtilisin/kexin type 9 enzyme inhibitors, PCSK9 inhibitors) were not prescribed despite his very high-risk profile. Therefore, the therapeutic targets set by clinical guidelines were not achieved for a long time.

## Case Presentation

**Figure 1:**
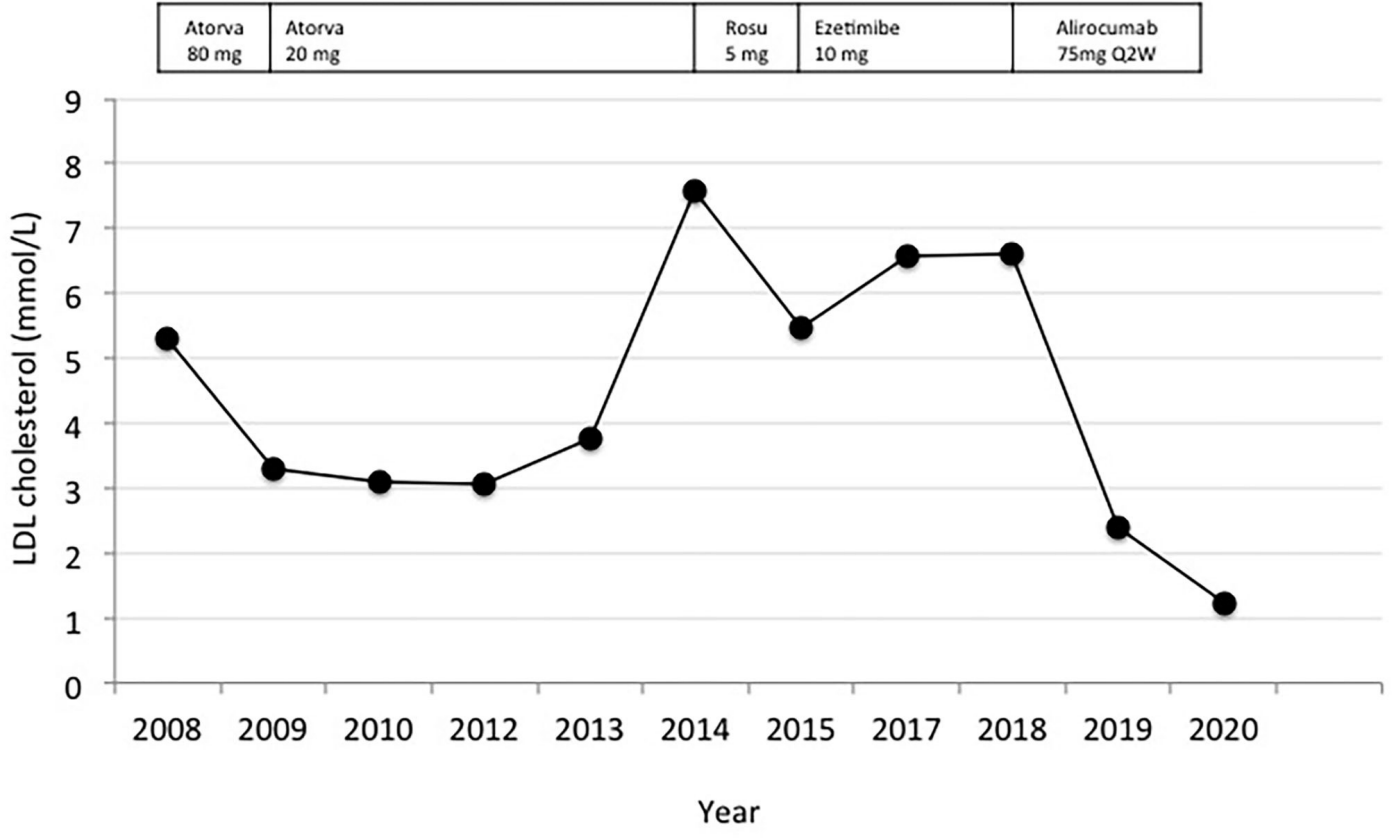
LDL Cholesterol levels between 2008 and 2020 and concomitant lipid lowering treatments. Atorva, atorvastatin; Rosu, rosuvastatin; Q2W, every 2 weeks.

In July 2018, an 80-year-old-man was referred to our Outpatient Lipid Clinic (Pisa University Hospital) for the management of hypercholesterolemia in a setting of a statin contraindication. The patient had a family history of premature coronary artery disease and hypercholesterolemia. He used to be a heavy cigarette smoker. His medical history included ischemic heart disease, systemic hypertension, hyperuricemia, impaired fasting plasma glucose, and chronic kidney disease (stage 3a). In 2008, at the age of 70 years, the patient presented to the Emergency Room with epigastric pain. The initial ECG showed ST segment elevation from V2 to V6, I and aVL, with ST depression in inferior leads. Impaired left ventricular systolic function (EF 35%) and 3-vessel coronary artery disease were detected by echocardiographic and angiographic examination, respectively. He was treated with percutaneous coronary angioplasty and drug-eluting stents. The patient's cardiovascular history was silent until his first heart attack in 2008, when he started therapy with atorvastatin 80 mg once daily. He subsequently underwent multiple coronary revascularization procedures following the onset of inducible myocardial ischemia (from 2008 to 2013). In 2014, upon starting treatment with rosuvastatin 5 mg once daily for LDL cholesterol (LDLc) level of 7.59 mmol/L, the patient was admitted to the Emergency Room of a local hospital for generalized myalgia and muscle weakness impairing mobility. He also reported oliguria with dark urine (“tea-color”) 2 days prior to hospital admission. Lab investigations revealed elevated creatine kinase (CK) levels, peaking at 6685 U/L (normal values <190 U/L), creatinine 123.8 μmol/L (normal values 61.9–106.1 μmol/L) and potassium 5.3 mmol/L (normal values 3.5–5.1 mmol/L). A urine dipstick was positive for myoglobinuria. No specific etiology was identified to account for the rhabdomyolysis other than statin therapy. Muscle biopsy was not performed, and rosuvastatin was withdrawn. Intravenous fluid administration was provided, however acute kidney injury developed with elevated potassium values that could not be controlled with i.v., insulin/dextrose infusion. Therefore, the patient underwent four dialysis sessions *via* a temporary vascular catheter. CK levels progressively declined (184 U/L) and the renal function improved so that the patient became dialysis-independent although residual impaired kidney function persisted (creatinine 150.3 μmol/L, stage 3b). Clinically, the patient reported a significant improvement in muscle symptoms, and he was discharged after a cycle of physiotherapy with a prescription of ezetimibe (10 mg daily) and diet with reduced daily intake of saturated- and trans-fatty acids. This treatment however failed to effectively reduce LDLc levels that ranged between 5.9 and 6.6 mmol/L for the ensuing 4-years (Figure 1).

In 2018, at the time of our evaluation, the patient was still on ezetimibe and lipid levels were: total cholesterol 9.5 mmol/L; LDLc 6.6 mmol/L; HDLc 1.3 mmol/L; non-HDL cholesterol 8.1 mmol/L, and triglycerides 3.5 mmol/L. Unfortunately, lipoprotein (a) [Lp(a)] levels were not assessed in our patient, although, Lp(a) is associated with a poor long-term prognosis in the elderly with ischemic heart disease and with an increased risk of stroke (8, 9). The physical examination was normal, according to the age. His temperature was 36.7°C, the pulse rate was 88 beats/min with a blood pressure of 140/80 mmHg on amlodipine (10 mg daily). On auscultation, a grade 2/6 systolic murmur was heard near the base of the heart. In consideration of the age, we performed a multidimensional geriatric assessment and results of the different fields of interest are shown in Table 1. The geriatric assessment showed good functional and mental status supporting the treatment intensification. Among the various drugs available, we excluded bile acid sequestrants, although, useful in the treatment of dyslipidemia (10) because of possible adverse effects, especially gastrointestinal, and interaction with other drugs that is a concern in the elderly frequently on polypharmacy. Therefore, we directed our choice toward the most modern approach available (i.e., proprotein convertase subtilisin-kexin type 9 inhibitors, PCSK9 inhibitors). Alirocumab 75 mg was prescribed subcutaneously once every 2 weeks as add-on to ezetimibe, without dose titration due to the patient's reluctance. The patient underwent clinical evaluation and lab test follow-up at 3–6-month interval for 24 months. At this time point, the multidimensional geriatric assessment showed no significant changes in functional status, comorbidity, cognition, mental health and nutrition (Table 1) and no major geriatric syndromes (i.e., delirium, falls, incontinence, pressure ulcers, and functional decline) or disability have developed. Alirocumab was well-tolerated and based on electronic prescription report, with 100% treatment adherence. No adverse effects were reported and LDLc progressively decreased to >50% from baseline after 3 months of treatment, (2.6 mmol/L). A downward trend was confirmed at 6 and 12 months (2.3 and 2.4 mmol/L, respectively). At 24 months, LDLc was 82% lower as compared to baseline values (from 6.6 to 1.2 mmol/L; Figure 1).

**Table 1:** Scores of the complete geriatric evaluation at baseline and at 24-month follow-up.

	**Scores at baseline**	**Scores at 24-month follow up**	**Reference values**
ADL^*^	6	6	From 0 (low function, dependent) to 6 (high function, independent)
IADL^**^	5	5	From 0 (low function, dependent) to 5 (high function, independent) for men
MMSE^°^	26.1	27.1	A score ≥24 was classified as normal
GDS^#^	4	5	A score >5 points was suggestive of depression
MNA^§^	28	27.5	A score ≥24 identified a normal nutritional status
TGUG^∧^	10	12	A time ≤ 12 s indicated the optimal cut-off time for performing the test

* *ADL: Activities of Daily Living.*

** *IADL: Instrumental Activities of Daily Living.*

° *MMSE: Mini Mental State Examination.*

# *GDS: Geriatric Depression Scale.*

§ *MNA: Mini Nutritional Assessment.*

∧ *TGUG: Timed Get-up-and-Go*.

## Methods

Triglycerides, total and HDL cholesterol were determined in fasting blood samples by colorimetric enzymatic methods; non-HDL cholesterol was calculated by the following formula: total cholesterol – HDL cholesterol. LDL cholesterol was calculated by the Friedewald formula. Serum creatinine was measured by the modified Jaffe method, traceable to IDMS, and eGFR was calculated by the CKD Epidemiology Collaboration equation. All other parameters were measured by standard method.

## Discussion

To the best of our knowledge, this is a unique case report illustrating the possibility to overcome therapeutic inertia in an octogenarian with severe hypercholesterolemia and ischemic heart disease. Our patient had a very high cardiovascular risk, yet his LDLc levels were poorly controlled. This was most likely the consequence of two aspects. The first refers to the fact that the patient and the physician had been reluctant to undertake potentially harmful treatment since severe rhabdomyolysis with rosuvastatin had already occurred. The second aspect could relate to the common feeling still existing within the medical community that an adequate reduction in LDLc levels may not be so relevant in the elderly and even more so in the case of treatment of hypercholesterolemia with high intensity drugs.

“Therapeutic inertia” is multifactorial in nature, but old age is a common contributing factor (11). However, although, older age is certainly associated with increased morbidity and mortality, chronological age it may be that relevant for many older people (12) as individuals age differently according to aging patterns also known as “ageotypes” (13). Lack of trials exclusively designed to include elderly people also contributes to therapeutic inertia (14). Although, cholesterol lowering has been proven to be essential for prevention and reduction of cardiovascular risk in a wide range of individuals (15), the general perception is that LDLc may not be so relevant in the elderly. As such, older subjects are less likely to receive proper lipid lowering treatment and much less likely to receive high-intensity lipid lowering therapy. The use of statins in the elderly, up to 75 years of age, with the aim of reducing LDLc is suitable in the case of patients on secondary prevention or at very high cardiovascular risk because of multiple concomitant risk factors, whereas it would not be justified, with few exceptions, in the case of patients >85 years of age (16). The clinical benefit of LDLc reduction in elderly patients remains debated. However, a recent meta-analysis has shown how in patients aged 75 years and older, lipid lowering is as effective in reducing cardiovascular events as it is in individuals younger than 75 years (17). These results strengthen guideline recommendations for the use of lipid-lowering therapies, including non-statin treatment, in older patients as well (18).

Common concerns about aggressive treatment in the elderly includes fears of adverse events, in particular muscle-related effects of statins, ranging from pains to rhabdomyolysis (19) as it was the case for our patient. As a result, treatment was limited to ezetimibe allowing no more than 15–20% reduction of LDLc levels. Nonetheless, treatment intensification with a PCSK9-inhibitor was not considered even though these agents have been available since 2015 for medical use and reimbursed in Italy since 2017, where the rate of prescription of PCSK9 inhibitors still appears below expectations (20). A sub-analysis of the ODYSSEY OUTCOMES, although, this trial was not specifically designed for the older population, showed that alirocumab improved outcomes irrespective of age (21) with no differences in adverse events between alirocumab and placebo. Another factor that may support therapeutic inertia with respect to PCSK9-inhibitors may be represented by cost (22). Altogether, these factors account for many patients to be excluded from effective treatment only based on their age. Appropriate and careful distinction between chronologic vs. physiologic age may provide sufficient background for a more personalized therapeutic approach. To this end, we applied a comprehensive geriatric evaluation establishing that the patient's physiological age and performance did not contraindicate the use of a treatment capable of achieving LDLc target levels. Therapeutic algorithms have been recently developed that could improve prescription performance if associated to a comprehensive geriatric evaluation (23). In our case, simple and validated tools have contributed to refine estimates of the risk and guide personalized treatment thus maximizing the likelihood of a positive outcome. The area of lipid-modulating agents is constantly evolving, and new pharmacological molecules (e.g., inclisiran and bempedoic acid) with novel mechanisms of action have recently been approved (24). Having new opportunities in the treatment of hypercholesterolemia could mark a major advance in the care of patients with high and very high-risk cardiovascular disease, and not only in statin-intolerant individuals. Adherence and persistence to optimal therapy could be improved as these new drugs are administered orally (bempedoic acid) or subcutaneously every 6 months (inclisiran). Whether the recent introduction of bempedoic acid or the introduction in the next few months of inclisiran may help overcoming therapeutic inertia in elderly people may be an option although it remains to be ascertained.

## Conclusions

In conclusion, although, this case report is limited in its applications to general patient care, it is instrumental in highlightening that even in older patients with dyslipidemia and high cardiovascular risk there is the need to reach therapeutic goals to ensure effective reduction of cardiovascular events. Misconceptions about age may limit implementation of appropriate treatment. PCSK9 inhibitors can provide effective and safe reduction of LDL-cholesterol in elderly patients with contraindication to the use of statins. To plan treatment and facilitate decisions, the clinician may rely on the first letters of the alphabet “ABC” as an acronym for “Always the Best Care,” also for the elderly.

## Data Availability Statement

The raw data supporting the conclusions of this article will be made available by the authors, without undue reservation.

## Ethics Statement

Written informed consent was obtained from the individual(s) for the publication of any potentially identifiable images or data included in this article.

## Author Contributions

AD, GD, GP, and RM have contributed to collection, analysis, and interpretation of data. AD and GP was involved in the management of the patient. AD performed the geriatric evaluation and wrote the first and final version of the manuscript. SDP overviewed the discussion. All authors have critically revised the article, approved of the final version of the manuscript, and made substantial contribution to the conception of the work.

## Conflict of Interest

SDP reports grants and personal fees from AstraZeneca, Boheringer Ingelheim, Novartis; and personal fees from Applied Therapeutics, Eli Lilly, GlaxoSmihKline, Novo Nordisk, Laboratoires Servier, Sanofi, and Takeda Pharmaceuticals. GD has received research funding from Eli Lilly and AstraZeneca. GP reports lecture fees from AstraZeneca, Boehringer Ingelheim, Eli Lilly, Merck Sharp & Dohme, Novo Nordisk, Sigma-Tau, and Takeda, and travel grants from AstraZeneca, Novo Nordisk, and Takeda. The remaining authors declare that the research was conducted in the absence of any commercial or financial relationships that could be construed as a potential conflict of interest.
